# Assessment of right ventricular remodeling in chronic thromboembolic pulmonary hypertension by 2D-speckle tracking echocardiography: A comparison with cardiac magnetic resonance

**DOI:** 10.3389/fcvm.2022.999389

**Published:** 2022-11-17

**Authors:** Yeqing Wang, Dichen Guo, Mingxi Liu, Xinyuan Zhang, Huimin Hu, Hao Yang, Yuanhua Yang, Xiuzhang Lv, Yidan Li, Xiaojuan Guo

**Affiliations:** ^1^Department of Echocardiography, Heart Center, Beijing Chao-Yang Hospital, Capital Medical University, Beijing, China; ^2^Department of Radiology, Beijing Chao Yang Hospital, Capital Medical University, Beijing, China; ^3^Department of Respiratory and Critical Care Medicine, Beijing Institute of Respiratory Medicine and Beijing Chao-Yang Hospital, Capital Medical University, Beijing, China

**Keywords:** chronic thromboembolic pulmonary hypertension, right ventricular remodeling, two-dimensional speckle-tracking echocardiography, cardiac magnetic resonance feature-tracking, echocardiography

## Abstract

**Background:**

Right heart remodeling occurs in a substantial proportion of patients with chronic thromboembolic pulmonary hypertension (CTEPH) and significantly affects their prognosis. Two-dimensional speckle-tracking echocardiography (2D-STE) can be used to evaluate myocardial deformation under physiological and pathological conditions. This study aimed to assess the feasibility of 2D-STE for evaluating right ventricular (RV) remodeling in CTEPH patients.

**Methods:**

This retrospective study included 21 CTEPH patients who underwent transthoracic echocardiography (TTE) and cardiac magnetic resonance (CMR). Data for the following parameters that can reflect RV function were collected: tricuspid annular plane systolic excursion (TAPSE), fractional area change (FAC), right ventricular index of myocardial performance (RIMP), peak systolic velocity of the tricuspid annulus (S'), and CMR-right ventricular ejection fraction (CMR-RVEF). The following strain parameters were calculated using post-processing software: STE-RV global longitudinal strain (STE-RVGLS), STE-RV free wall longitudinal strain (STE-RVFWLS), and CMR-RVGLS.

**Results:**

As CMR-RVEF deteriorated, RV remodeling in CTEPH patients became more apparent and was mainly characterized by significant enlargement of the RV, weakening of myocardial deformation, and a decrease in RV contractility (RV area, STE-RVFWLS, STE-RVGLS: mild vs. severe and moderate vs. severe, *p* < 0.05; CMR-RVGLS: mild vs. severe, *p* < 0.05; TAPSE: moderate vs. severe, *p* < 0.05). Moreover, the Pearson correlation coefficient for correlation with CMR-derived RVEF was stronger for RVFWLS than for CMR-GLS (*r*-value: 0.70 vs. 0.68), and the strain values measured by 2D-STE showed a weak correlation with right heart catheterization data. Bland-Altman analysis showed good agreement between 2D-STE and CMR-feature tracking (FT) for RVGLS (bias = −0.96; 95% limit of agreement from −8.42 to 6.49).

**Conclusions:**

For the measurement of RVGLS, 2D-STE is similarly feasible to CMR-FT and could sensitively identify right heart remodeling.

## Introduction

Chronic thromboembolic pulmonary hypertension (CTEPH) is a common and significant type of PH characterized by increased pulmonary vascular resistance due to progressive pulmonary vascular remodeling (1). As the disease progresses, the patient's right ventricular (RV) function decreases, resulting in right-sided heart failure and even death. The increase in pulmonary vascular resistance caused by progressive pulmonary vascular remodeling may lead to right heart failure. Previous research showed that RV function is an independent prognostic factor in patients with PH (2, 3), and the long-term prognosis of patients with World Health Organization (WHO)-Functional Class (FC) III/IV grade pulmonary arterial hypertension is poor (4). Remodeling in the right heart plays an important role in the progression and prognosis of CTEPH patients (5) and reflects alteration of RV structure, function, and hemodynamics. Therefore, the ability to accurately evaluate RV remodeling is particularly important. Quantitative assessment of RV function remains challenging, though, due to the complex geometry of this structure and the different orientations of myocardial fibers. Currently, the RV ejection fraction (RVEF) measured by cardiac magnetic resonance (CMR) imaging is considered the imaging reference standard for the assessment of RV systolic function (6), but clinical application of this technique is limited due to the substantial time requirement and high cost. In addition, in the early stage of the disease, the RVEF is not sensitive to changes in cardiac function.

In view of the above-mentioned reasons, strain parameters that can quantify RV deformation were developed through the post-processing of images by the derivation technique. Strain refers to the degree of myocardial deformation in the specified direction from the original shape (end of diastole) to the minimal length (end of systole) and is expressed as a percentage, reflecting the contractile force of myocardial fibers in different directions. Previous research has confirmed that strain is also associated with clinical outcomes in patients with PH, suggesting that it could be used as a standard for quantifying RV function (7, 8). In recent years, the application of 2D-STE and CMR-feature tracking (FT) for measuring myocardial strain has been recognized as more valuable and superior to conventional Doppler echocardiography for evaluating RV function (9–11). The two technologies were also used to evaluate RV function in patients and healthy people with arrhythmogenic right ventricular cardiomyopathy (ARVC) patients and healthy people. In these patients, the two techniques showed good correlation and agreement (12, 13).

The aims of the present study were twofold. First, we used two different algorithms to analyze RV strain parameters to evaluate the agreement and correlation between 2D-STE and CMR-FT. Second, we divided the research population into three groups according to the degree to which RVEF was reduced to verify the ability of 2D-STE to identify RV remodeling in CTEPH patients. Our research results provide insight into expanding the theoretical basis for the use of imaging techniques to guide treatment planning and prognosis prediction for CTEPH patients.

## Materials and methods

### Study population

This retrospective study included a total of 34 patients who had been diagnosed with CTEPH in Beijing Chaoyang Hospital from December 2016 to June 2021. All patients underwent transthoracic echocardiography (TTE) and CMR examination within a median interval of 5 days. The diagnosis of CTEPH was based on the patient's medical history, TTE, right heart catheterization (RHC), multidetector computed tomography (MDCT), and lung ventilation-perfusion scintigraphy according to established guidelines (1). Demographic data, baseline clinical characteristics, and hemodynamic measurements were obtained from electronic health records. The exclusion criteria were as follows: persistent atrial fibrillation or WHO FC grade IV, poor acoustic window, incomplete clinical data, and an interval of more than 1 month between TTE and CMR. The final study population included 21 patients. All procedures complied with the 1975 Declaration of Helsinki and were approved by the Ethics Committee of Beijing Chaoyang Hospital. Informed consent was obtained from each patient.

### Echocardiography

All patients underwent TTE examination using a Philips EPIQ 7C (Philips Healthcare, MA, USA) and a Doppler ultrasound machine equipped with an X5-1 probe (1–5 MHz). After optimizing the sector size, gain, and depth, an RV-focused four-chamber(4Ch) view was acquired at a heart rate of 71 (±8) bpm. Data measurements were based on the guidelines of the American Society of Echocardiography (14). Conventional parameters that reflect RV function include tricuspid annular plane systolic excursion (TAPSE), peak systolic velocity of the tricuspid annulus (S'), RV fractional area change (RVFAC), and RV index of myocardial performance (RIMP), which was calculated using the formula: RIMP = (isovolumetric contraction time + isovolumetric relaxation time)/ejection time. Left ventricular ejection fraction (LVEF) was measured by Simpson's method. The estimation of pulmonary artery systolic pressure (sPAP) was based on the tricuspid regurgitation peak velocity and right atrial pressure (based on inferior vena cava diameter and inspiratory collapse rate).

### 2D-STE

Based on conventional apical four-chamber, two-chamber, and three-chamber views with a frame rate of 60–80 frames/s, images from five consecutive cardiac cycles were recorded in DICOM format, and offline analysis was performed using QLAB version 13.0 (Philips, Andover, MA). The software then automatically tracked a region of interest, and if necessary, manual adjustment was performed to obtain the best image. Finally, the RV global longitudinal strain (RVGLS) and RV free wall longitudinal strain (RVFWLS) were obtained. All strain measurements were performed by an experienced physician (Wang) blinded to all clinical data.

### CMR

All patients underwent CMR scanning on a 3.0-T scanner (Prisma, Erlangen, Siemens Healthcare, Germany) with a body-flex receiver coil following an ECG examination and breath-hold technique. After scout imaging, 8–12 continuous short-axis plane images were acquired to cover both ventricles from base to apex using Ture fast imaging with a steady-state precession sequence. The MR parameters used were: repetition time of 3.0–3.2 ms, echo time of 1.4 ms, flip angle 70°, the field of view 320 × 360 mm, matrix size 256 × 256 mm, slice thickness of 8 mm, and 25 phases per cardiac cycle and images along the long-axis plane were acquired in the four-, three- and two-chamber views as well.

### CMR-FT

CMR image analysis was performed offline by two experienced observers (MX Liu and XJ Guo) using commercial software (CMR42 version 5.11.3, Circle Cardiovascular Imaging, Calgary, Alberta, Canada). The endocardial and epicardial traces were processed automatically in the serial short-axis plane images at the end-diastolic and end-systolic phases. The results were derived by excluding the papillary muscles and moderator bands. First, end-diastolic volume (EDV), end-systolic volume (ESV), and RVEF were calculated using a cardiac function analysis model; then, myocardial strain analysis was performed using a tissue tracking model importing long-axis slices (15). The global and regional (apical, mid-ventricular, and basal) feature tracking parameters were calculated automatically, including myocardial longitudinal peak strain (PS), systolic peak strain rate (SPSR), and diastolic peak strain rate (DPSR). PS was defined as the maximum absolute value of the strain in the entire cardiac cycle; SPSR was defined as the maximum absolute value of the systolic strain rate in one cardiac cycle, and DPSR was defined as the maximum absolute value of the diastolic strain rate in one cardiac cycle.

### Statistical analysis

Normal data distribution was tested using the one-sample Shapiro–Wilk test, and all the data are expressed as absolute values. Normally distributed data are expressed as mean ± standard deviation (SD), and non-normally distributed data are presented using median and interquartile range (IQR) values (expressed in percentage). According to established guidelines (16, 17), abnormal RV dysfunction was defined as RVEF < 45% measured by CMR and further classified as mildly decreased (RVEF 36–45%), moderately decreased (RVEF 24–36%), or severely decreased (RVEF < 24%). Differences between cohorts were assessed for statistical significance using analysis of variance (ANOVA) and the Kruskal-Wallis H test, with subsequent Bonferroni *post-hoc* correction for multiple comparisons. Correlations between 2D-STE and CMR-FT findings were evaluated using Pearson correlation coefficients. Agreement between the two techniques was assessed by Bland-Altman analysis [bias and 95% limit of agreement (LOA)]. All statistical analyses were performed using Prism 8.0 software (GraphPad, La Jolla, CA) and SPSS version 26.0 (SPSS, Inc., Chicago, IL, USA). Differences were considered significant if *p* < 0.05.

## Results

### Clinical and hemodynamic data

Of the 34 patients with CTEPH initially identified, we excluded eight patients due to poor image quality, three patients due to incomplete clinical information, one patient with WHO-FC IV PH, and one patient for whom more than 1 month passed between the two examinations. The baseline clinical characteristics and hemodynamic data of the 21 patients included in the final analysis are presented in Table 1. The patients had a median age of 63 years; 52% were male, and 48% had WHO FC II disease. All patients had normal left heart function (LVEF 64.6 ± 3.9%), and the mean pulmonary arterial pressure (mPAP) measured by RHC was 51.7 ± 12.0 mmHg. As indicated by CMR-RVEF, RV function was decreased to varying degrees (29.7 ± 10.0%) in the study population.

**Table 1:** Demographic, clinical, and hemodynamic characteristics at baseline.

**Characteristic**	**Patients (*n* = 21)**	***p*-value**
Age, years	63 (13)	0.008
Male, *n* (%)	11 (52)	–
Systolic BP, mmHg	116 (29)	0.014
Diastolic BP, mmHg	73 (18)	0.018
Heart rate, beats/min	78.9 ± 12.3	0.532
BSA, cm^2^	1.8 (0.3)	0.007
WHO FC		–
I	4 (19)	
II	10 (48)	
III	7 (33)	
IV	0 (0)	
**Echocardiographic measurements**		
LAAPd, mm	36.4 ± 5.4	0.739
LATd, mm	36 (4.5)	0.001
LASId, mm	49 (4)	0.005
LV end-diastolic diameter, mm	41.5 ± 5.1	0.308
LV end-systolic diameter, mm	26 (3.5)	0.001
IVST, mm	9 (1)	0.004
LVPWT, mm	9 (2)	0.008
E velocity, m/s	50 (22.5)	0.010
A velocity, m/s	66.4 ± 17.5	0.465
E/A ratio	0.7 (0.3)	0.000
LVEF, %	64.6 ± 3.9	0.831
**CMR measurements**		
RVEDV, mL	190.8 ± 37.5	0.852
RVESV, mL	134.4 ± 37.7	0.641
RVEF, %	29.7 ± 10.0	0.059
**Hemodynamic data**		
mPAP, mmHg	51.7 ± 12.0	0.747
PCWP, mmHg	9 (5.5)	0.022
CI, L/min per m^2^	2.2 ± 0.4	0.808
PVR, Woods units	11.5 ± 5.5	0.069

Data are expressed as mean ± SD, median (IQR), or n (%).

BP, blood pressure; BSA, body surface area; WHO FC, World Health Organization Functional Class; LAAPd, left atrial anteroposterior diameter; LATd, left atrial transverse diameter; LASId, left atrial superoinferior diameter; IVST, interventricular septal thickness; LVPWT, left ventricular posterior wall thickness; LVEF, left ventricle ejection fraction; RVEDV, right ventricular end-diastolic volume; RVESV, right ventricular end-systolic volume; mPAP, mean pulmonary artery pressure; PCWP, pulmonary capillary wedge pressure; CI; cardiac index; PVR, pulmonary vascular resistance.

### Comparison among subgroups with differing degrees of RV dysfunction

In this study, CMR-RVEF was used as the imaging gold standard to reflect patients' RV systolic function, and patients were divided into three groups according to the CMR-RVEF value. Among them, RVEF 36–45% represented a mild decrease in RV function (*n* = 7), RVEF 24–36% showed a moderate decrease in RV function (*n* = 7), and RVEF < 24% showed a severe decrease in RV function (*n* = 7). The results of comparisons among these groups are presented in Table 2. Overall, in terms of conventional parameters, compared with the patients with a mild or moderate decrease in RVEF, the patients with severe RV dysfunction had higher RV end-diastolic area (EDA) and RV end-systolic area (ESA) (mild vs. severe, moderate vs. severe, all *p* < 0.05), which reflected structural RV remodeling with the progression of the disease. Additionally, TAPSE, a commonly used echocardiographic index reflecting RV systolic function, differed significantly between patients with moderate and severe RVEF reduction, while the other parameters showed no statistically significant difference between groups. Regarding strain values obtained from post-processing software, the RV strain measured by 2D-STE was higher than that measured by CMR-FT. Similarly, as the RVEF decreased, the strain values assessed by these two techniques decreased significantly. Significant differences were observed between cohorts (STE-FWLS, STE-GLS: mild vs. severe, moderate vs. severe; CMR-GLS: mild vs. severe, all *p* < 0.05). With regard to hemodynamic parameters, although no significant differences were detected between groups, a specific trend could still be seen.

**Table 2:** Comparison of clinical data among CTEPH patients with different degrees of RV dysfunction.

**Variables**	**Mild RV dysfunction (RVEF 36–45%), *n* = 7**	**Moderate RV dysfunction (RVEF 24–36%), *n* = 7**	**Severe RV dysfunction (RVEF < 24%), *n* = 7**
Age, years	66.1 ± 7.1	59.7 ± 10.8	55.9 ± 13.8
**Echocardiographic measurements**			
EI	1.4 ± 0.2	1.3 ± 0.2	1.4 ± 0.1
D_MPA_, mm	31.1 ± 4.0	32.0 ± 4.0	37.0 ± 6.3
RV basal diameter, mm	44.4 ± 4.4	46.1 ± 4.8	50.6 ± 5.7
RV EDA, cm^2^	25.2 ± 6.3	26.8 ± 3.6	34.0 ± 3.0^**#⋆**^
RV ESA, cm^2^	17.6 ± 6.8	19.0 ± 3.4	25.4 ± 4.5^**#⋆**^
PASP, mmHg	86.0 ± 28.6	94.0 ± 12.9	103.9 ± 11.6
TAPSE, mm	15.7 ± 5.0	16.2 ± 1.9	12.9 ± 1.5^**⋆**^
S', cm/s	9.1 ± 1.6	10.6 ± 3.4	9.4 ± 1.1
FAC, %	32.7 ± 12.4	29.3 ± 6.1	25.6 ± 7.7
RIMP	0.8 ± 0.2	0.7 ± 0.1	0.9 ± 0.2
**Strain parameters**			
STE-RVFWLS, %	17.5 ± 5.2	17.2 ± 3.3	11.6 ± 1.9^**#⋆**^
STE-RVGLS, %	14.3 ± 4.7	13.6 ± 2.6*	9.4 ± 1.7^**#⋆**^
CMR-GLS, %	14.9 ± 5.1	11.6 ± 3.3*	8.0 ± 2.3^**#**^
**RHC**			
mPAP, mmHg	43.1 ± 10.1	54.7 ± 9.4	57.3 ± 12.4
PCWP, mmHg	8.7 ± 3.7	11.0 ± 5.2	8.9 ± 1.8
CI, L/min per m^2^	2.3 ± 0.6	2.4 ± 0.2	2.0 ± 0.4
PVR, Woods units	10.1 ± 5.7	10.2 ± 2.5	14.3 ± 6.9

Data are expressed as mean ± SD. Reported p-values refer to global p obtained with ANOVA and Kruskal–Wallis H test.

EI, eccentric index; D_MPA_, the diameter of main pulmonary artery; RV EDA, right ventricular end-diastolic area; RV ESA, right ventricular end-systolic area; PASP, pulmonary artery systolic pressure; TAPSE, tricuspid annular plane systolic excursion; S', peak systolic velocity of the tricuspid annulus; FAC, fractional area change; RIMP, right ventricular index of myocardial performance; STE-RVFWLS, speckle-tracking echocardiography-RV free wall longitudinal strain (STE-RVFWLS); STE-RVGLS, STE-RV global longitudinal strain; CMR-RVGLS, cardiac magnetic resonance-RVGLS; mPAP, mean pulmonary artery pressure; PCWP, pulmonary capillary wedge pressure; CI; cardiac index; PVR, pulmonary vascular resistance.

Bold values indicate statistical significance (p < 0.05). ^*^p < 0.05 for mild vs. moderate; ^#^p < 0.05 for mild vs. severe; ⋆p < 0.05 for moderate vs. severe.

**Figure 1:**
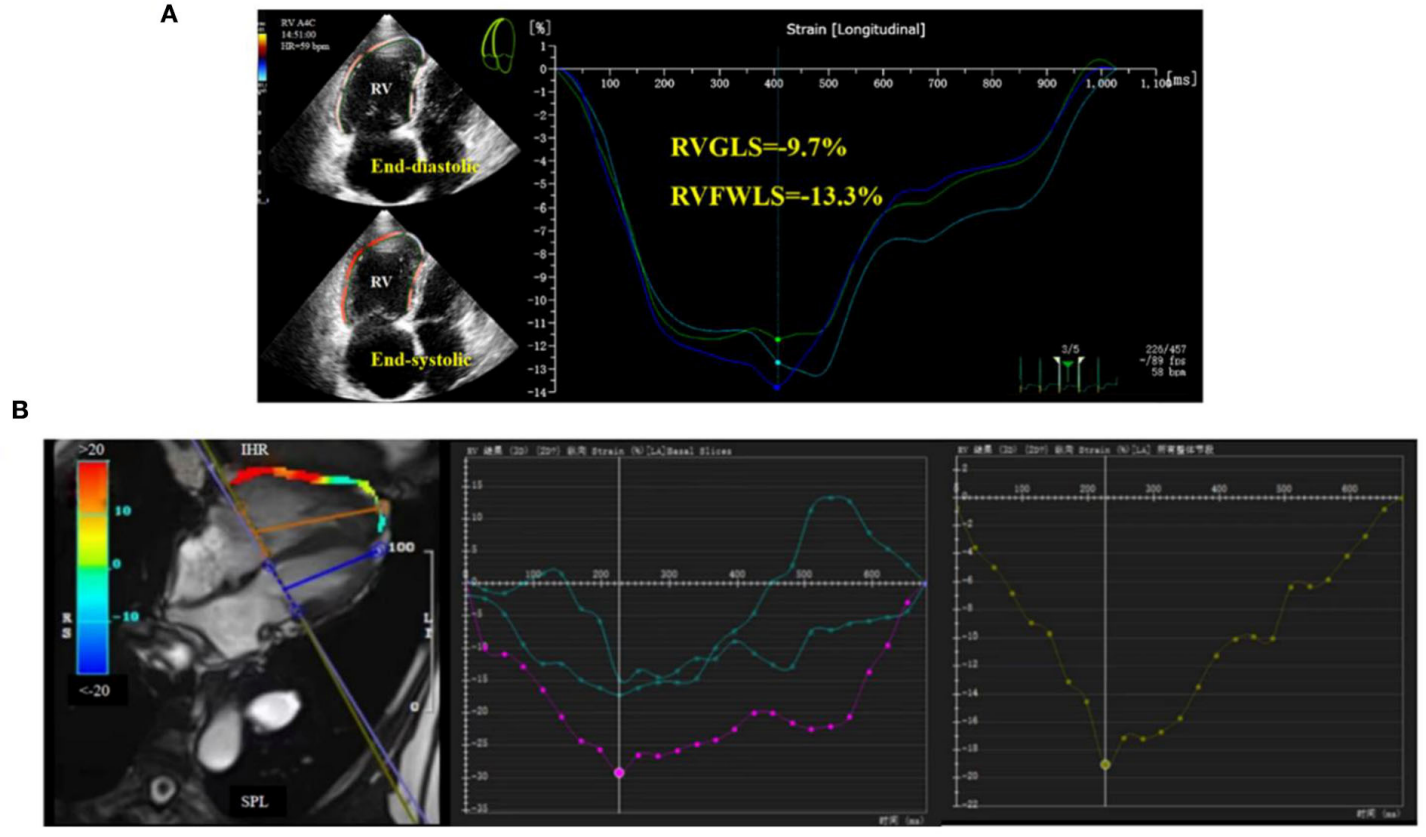
Right ventricular global longitudinal strain (RVGLS) determination in CTEPH patients. **(A)** 2D-STE for measurement of RVGLS, and **(B)** CMR-FT for measurement of RVGLS.

Subsequently, we further divided the study population into two cohorts according to WHO FC: WHO I/II and WHO III. Only CMR-GLS was found to differ significantly between the two groups. Due to the lack of clinical data for some patients, no significant differences were detected for most parameters, but the overall trends for numerical changes were as expected (data not shown). An example of RV strain measurement using post-processing of 2D-STE and CMR-FT images of a CTEPH patient is presented in Figure 1.

### Myocardial strain evaluation using 2D-STE compared to CMR-FT

**Figure 2:**
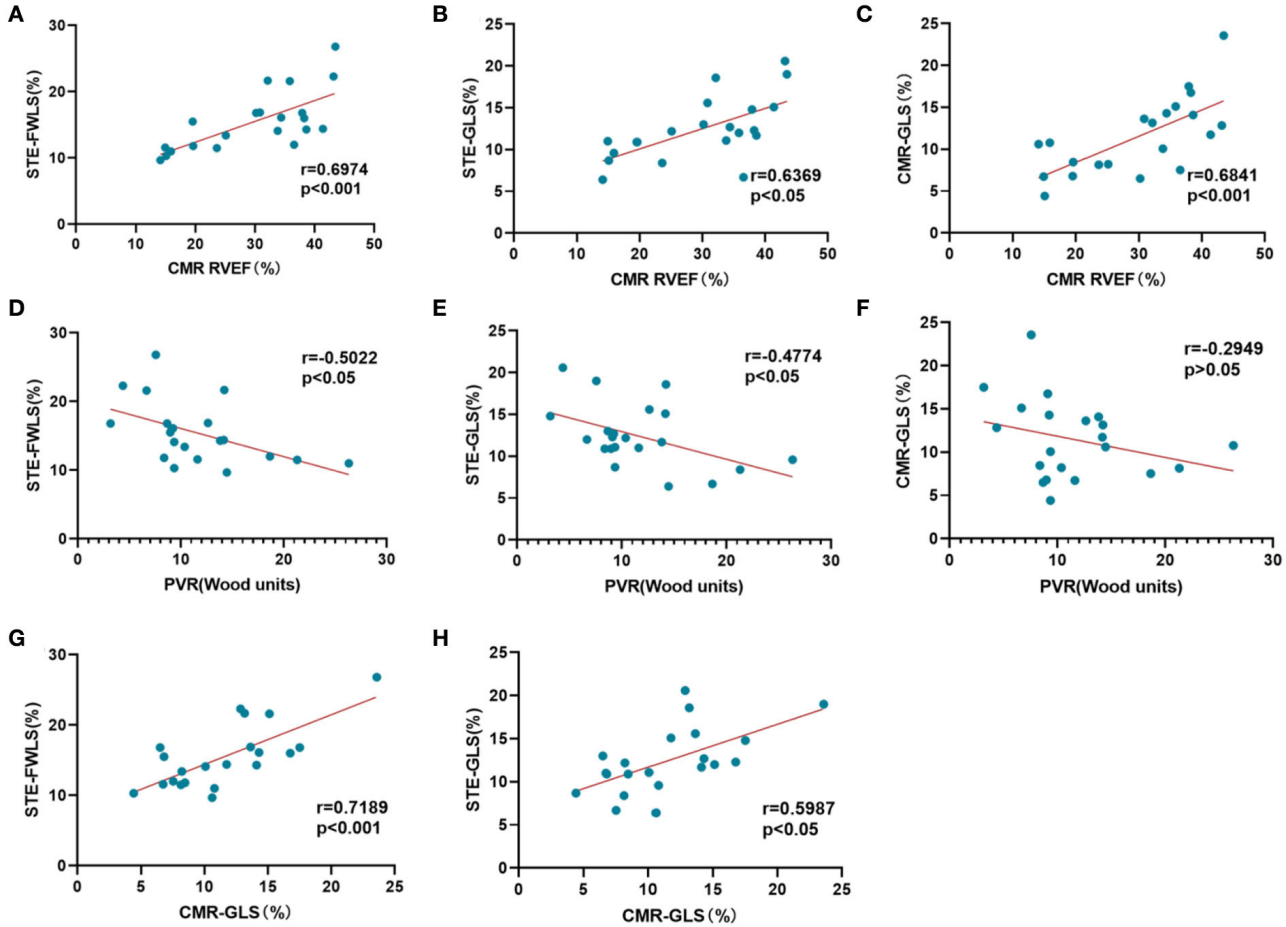
Correlation analysis of myocardial strain evaluation. **(A–C)** Correlation of STE-RVFWLS, STE-RVGLS, and CMR-RVGLS with CMR-RVEF. **(D–F)** Correlation of STE-RVFWLS, STE-RVGLS, and CMR-RVGLS with PVR. **(G,H)** Correlation of STE-FWLS and STE-GLS with CMR-GLS.

Figure 2 demonstrates the correlations between strain, CMR-RVEF, and RHC data. In general, RVFWLS and RVGLS measured by 2D-STE were significantly correlated with CMR-GLS (*r* = 0.72, *p* < 0.001; *r* = 0.60, *p* < 0.05). Taking CMR-RVEF as the imaging gold standard to define RV systolic function, the results showed that STE-FWLS obtained from 2D-STE post-processing had the strongest correlation with CMR-RVEF (*r* = 0.70, *p* < 0.001), followed by CMR-GLS (*r* = 0.68, *p* < 0.001) and STE-GLS (*r* = 0.64, *p* < 0.05). Surprisingly, we also found weak correlations of STE-FWLS and STE-GLS with pulmonary vascular resistance (PVR) (*r* = −0.50, *p* < 0.05; *r* = −0.48, *p* < 0.05), while a similar result was not found for CMR-GLS (*r* = −0.29, *p* > 0.05).

### Agreement between STE and CMR-FT analysis of RV function

**Figure 3:**
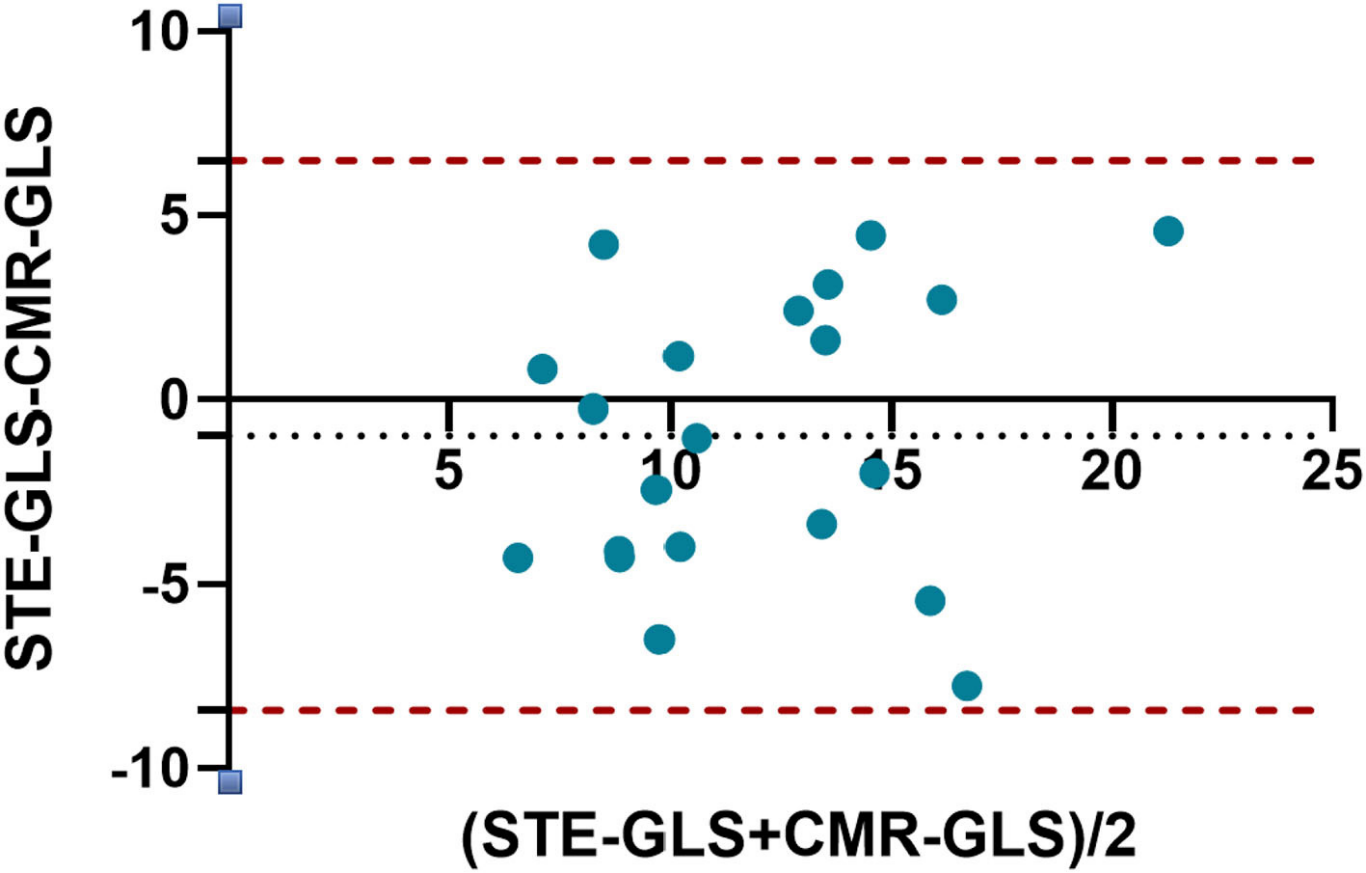
Bland-Altman plot for agreement between RVGLS obtained by 2D-STE and CMR-FT. The horizontal dots represent the mean difference, and the dashed lines represent ±2 standard deviations of the difference.

Bland-Altman plots showed that RVGLS values obtained from 2D-STE and CMR-FT were in good agreement (bias: −0.9648), and all points were within the 95% LOA (lower LOA: −8.422 and upper LOA: 6.493; Figure 3). The mean RV LS obtained from 2D-STE was higher than that determined from CMR-FT (12.4 ± 3.8 vs. 11.5 ± 4.6, respectively).

## Discussion

The objectives of the present study were to verify the feasibility of 2D-STE for identifying RV remodeling in CTEPH patients and to analyze the agreement and correlation of 2D-STE measurements with those obtained by CMR. The results of this study demonstrate that: (i) 2D-STE can sensitively recognize right heart remodeling; (ii) compared with conventional parameters, the strain value can better reflect impaired RV function; and (iii) in patients with CTEPH, good agreement was observed between 2D-STE and CMR-FT for global LS quantification.

CTEPH is caused by repeated pulmonary embolism due to incomplete dissolution of thrombus or repeated shedding of deep vein thrombus in the lower limbs and gradually develops into chronic pulmonary embolism (1). CTEPH gradually causes structural and physiological changes in the right ventricle as the disease progresses. The patient eventually experiences right heart failure, which is the most common cause of death in these patients. RV remodeling, defined by changes in the morphology, structure, and function of myocardium (18, 19), is a consequence of disease progression. Recent guidelines emphasize that right heart function is important in determining the severity and clinical outcome of CTEPH (1). Thus, an examination method for accurate and regular monitoring of the degree of right heart remodeling is urgently needed.

According to the 2022 ESC/ERC guidelines, echocardiography is still the most widely used non-invasive diagnostic tool to evaluate PH. It is clinically regarded as the primary choice for evaluating RV systolic function (1). Traditional echocardiographic parameters are insensitive to subtle structural and functional changes. RVEF is considered the imaging gold standard but is not sensitive to early diastolic dysfunction. With the development of 2D-STE and CMR-FT, strain measurements may provide more accurate quantification of RV function and potentially additional information for clinical use with good repeatability and high sensitivity. The strain parameters measured by 2D-STE reflect the motion of the myocardium in different directions and segments, which can objectively quantify myocardial deformation in an angle-independent way. This relatively new technology has been widely studied for its ability to evaluate RV function in patients with different types of heart diseases (20, 21). Although EF is an index of radial function and strain assesses longitudinal function, many studies have confirmed the correlation between strain and EF, and strain is more sensitive than EF in identifying early myocardial injury (1, 22). Moreover, because CMR-FT adopts an algorithm different from 2D-STE and can also quantify the change in myocardial motor function; thus, such measurements have been gradually accepted as superior to RVEF (23). At present, CMR-FT has been mainly used in evaluating left heart disease. Our study excluded cases with poor-quality images, and the entire strain measurement process was carried out by a physician who was skilled in operating the software to ensure the results were reliable.

RV remodeling is common in CTEPH. In this research cohort, the RV was enlarged with a decrease in RVEF, as reflected by the significant increases in RV EDA and RV ESA. Likewise, in the population with a severe reduction in CMR-RVEF, TAPSE also showed a statistically significant change. However, the same results were not found for other parameters, which may be related to the small size of the study population. Additionally, traditional echocardiographic parameters have not been sensitive enough to reflect local changes in the myocardium. Compared with conventional parameters, STE-FWLS and STE-GLS were decreased in the early stage of the disease. The strain values were significantly reduced with the reduction of RV systolic function, indicating that 2D-STE could sensitively detect the damage of myocardial deformation and be advantageous for identifying early RV remodeling. On the other hand, comparing the two imaging techniques showed good correlation and agreement between strain measurements derived from 2D-STE and CMR-FT. However, further research is still needed to determine whether the two techniques can replace or complement each other. Notably, the strain values determined by 2D-STE were statistically correlated with PVR. As an invasive examination method, the evaluation of PVR by RHC has been confirmed by several studies to be related to the long-term prognosis of patients with different types of PH (24, 25), which brings new ideas for our research. We also divided the patients into two subgroups according to WHO FC (WHO I/II and WHO III). Although we found that, except for CMR-RVGLS, the parameters did not differ significantly between the two subgroups, we could still observe changing trends for the overall parameters. The lack of statistical significance for these findings may be due to the small number of patients in the study population. In our future research, we will expand our sample size for statistical analysis.

To our knowledge, previous studies have been conducted to validate STE compared to FT in patients with arrhythmogenic right ventricular cardiomyopathy, hypertrophic cardiomyopathy, and aortic valve stenosis (13, 26, 27), and healthy people (28). Until now, only a few studies analyzing the RV strain of patients with CTEPH were determined by either of the two advanced techniques. Our research showed strong correlations of CMR-GLS, STE-FWLS, and STE-GLS with CMR-RVEF among all included parameters, which is consistent with the previous results (29). Additionally, similar to our findings, a previous study also found a high correlation between RVFWLS and CMR-RVEF, which reinforced the diagnostic value of this non-geometric index for RV systolic function (30). In a previous study of patients with heart failure, RVFWLS also demonstrated its prognostic value and improved risk stratification (31). However, another study found that the correlation between STE-GLS and CMR-RVEF was better than that of STE-FWLS with CRM-RVEF, which may be related to the use of different post-processing software and differences in the study populations (32). Finally, CMR-FT produced a significantly lower strain value than STE. This phenomenon was in accordance with previous findings comparing STE to tagging (28, 33). This result may be related to the fact that the time resolution of CMR is lower than that of ultrasound.

### Clinical implications

For clinicians, choosing a convenient and accurate method for evaluating RV function in CTEPH patients is of great significance, not only for diagnosing the disease but also for individualized treatment. The present study showed that 2D-STE could sensitively identify right heart remodeling and showed similar feasibility to CMR for assessing RV deformation. Considering that 2D-STE is easier and more economical in routine echocardiography and can be performed in most hospitals and that there are no guidelines providing normal reference ranges for the whole and segmental RV strain from CMR-FT, we propose that when CMR is not available, STE-RVFWLS can replace CMR-RVEF as an indicator of RV function.

### Study limitations

This study has several limitations. Firstly, this was a single-center, retrospective study with a small number of patients. Secondly, to our knowledge, the pathogenesis of CTEPH differs from other types of PH, and whether these results can be applied to other types of PH requires further study. Moreover, CMR and TTE were not carried out simultaneously. However, all patients maintained stable clinical conditions between the imaging examinations, and no significant volume changes were recorded between the two assessments. Future research with a larger study population must confirm our findings to address these limitations. As far as we know, the assessment of myocardial strain includes longitudinal and radial components and circumferential components. In the future, we will further evaluate myocardial motion in multiple directions to verify echocardiography's role in predicting the prognosis of PH. Although our results showed high reproducibility between the two post-processing methods, additional research is needed to confirm whether the measurement results can be used interchangeably.

## Conclusions

In conclusion, 2D-STE can sensitively identify the degree of RV remodeling in patients with CTEPH. Among the analyzed strain parameters, the present study demonstrated that RV strain values determined by 2D-STE showed a better non-geometric echocardiographic index and a strong correlation with CMR-RVEF. We also observed excellent reproducibility between 2D-STE and CMR-FT for determining RV strain values. Our results indicate that 2D-STE can offer a useful tool for the rapid and accurate evaluation of RV function in CTEPH.

## Data availability statement

The original contributions presented in the study are included in the article/supplementary material, further inquiries can be directed to yeqingwang2021@163.com.

## Author contributions

YL and XG contributed to conception and design of the study. YY and XL organized the database. XZ and HH performed the statistical analysis. YW wrote the first draft of the manuscript. ML, HY, DG, and YW wrote sections of the manuscript. All authors contributed to manuscript revision, read, and approved the submitted version.

## Funding

The study was funded by the National Natural Science Foundation of China (81871356) and the Beijing Municipal Administration of Hospitals Incubating Program (PX2019013).

## Conflict of interest

The authors declare that the research was conducted in the absence of any commercial or financial relationships that could be construed as a potential conflict of interest.

## Publisher's note

All claims expressed in this article are solely those of the authors and do not necessarily represent those of their affiliated organizations, or those of the publisher, the editors and the reviewers. Any product that may be evaluated in this article, or claim that may be made by its manufacturer, is not guaranteed or endorsed by the publisher.
